# Transactions of the Medico-Chirurgical Society of Edinburgh

**Published:** 1835-04-01

**Authors:** 


### Transactions of the Medico-chirurgical Society of Edinburgh. Fasciculus the Second. January, 1835.

We think the Medico-Chirurgical Society of modern Athens has judged wisely in publishing their Transactions through the medium of our highly-respected contemporary of the North, instead of running the risk and incurring the trouble of a separate publication. The London—we beg pardon—the Royal Medico-Chirurgical Society are differently circumstanced. The Transactions are furnished to the members gratuitously, on account of their heavy annual subscriptions, and the public takes off a number of copies, sufficient, in general, to defray the expences of paper and type. A great circulation is thus ensured to the metropolitan Transactions, independently of the reviews and analyses of the particular articles in the various medical journals. There is only one inconvenience attending the plan of the Scotch Society—namely, that papers thus published are not so generally noticed in the periodicals, as if they appeared in a volume of original matter. When, however, the wide circulation of our contemporary is considered, the contributors have little to complain of—especially as our own journal and some others will give them an occasional lift. In the present article, we shall run over the papers in the order in which they stand, noticing lightly their more prominent features.

### I. HEMICRANIA. By Mr. BROWN.

Two cases are related. The first patient had hemicrania of the right side of the head exactly. After lasting for some time, a discharge of blood took place, with purulent matter, from the right ear and nostril. The relief was immediate and permanent. We have no doubt that local inflammation about the frontal sinuses, the ear, and the nasal fossæ, is often the cause of hemicrania, and other forms of headache.

The second case was that of a young man, rather dyspeptic, who applied to Mr. Brown, in January, 1834, for pain in the right eyebrow, slightly affecting his vision. There was some swelling and redness of the integuments. A poultice was applied, but without benefit, as were leeches. The headache was worse every morning, and the digestive organs seemed disordered. An emetic, purgative, and quinine were prescribed. Notwithstanding these, the attack came on at 4 o'clock every morning—gradually, for three hours, and then declined as gradually. In the afternoon, he felt quite well. The tonic treatment made no impression on the complaint. A grain of opium, given an hour before the paroxysm, moderated the attack, and next day stopped it entirely. Although these complaints are generally dependent on constitutional disorder, still there must be a local affection of nerve also—and this local affection deserves attention. There is, perhaps, always some topical irritation or inflammation.

### II. CASES OF INFLAMMATION OF THE ORBITAL LININGS. By Mr. CRAIG.

The first case was that of a female, aged 33, who complained of agonizing and deep-seated pain in the left eye, or rather orbit, extending to the head, and producing delirium at night. The conjunctiva was inflamed, and the eyeball prominent—iris slightly contractile—vision impaired—pulse full, and 120. Bled from the arm to 35 ounces—six leeches—calomel and purgatives. Next day the local symptoms were aggravated. Third day, stationary—fourth and fifth days, no relief—eyeball more protruded. Temporal arteriotomy to 12 ounces—leeches repeated—blisters—scarifications of conjunctiva—antimonials. Sixth day, the pain excruciating, and the patient re-applied the leeches herself. Eighth day, the eyeball was dreadfully protuberant—pulse small and feeble—sense of pulsation in the orbit. Ninth day, an abscess burst through the upper eyelid, and discharged healthy pus. The symptoms all became alleviated, and she soon recovered.

In the second case, a female was seized with severe pain in the left orbit, fever, delirium, prominence of eye, lachrymation. She was freely purged—leeched—cold applications. The pain moderated, but the eye became more prominent, and an abscess pointed towards the inner canthus, which was opened by Mr. Liston, and discharged a thick caseous matter. The case terminated favourably, though slowly.

### III. CASE OF HYDROCEPHALUS. By Dr. BALFOUR.

This case is interesting. The subject of it was a child, 13 months old, who was seized with convulsions the preceding evening, and which had continued to recur at short intervals. Dr. B. checked the convulsive fit, which he witnessed, by "the warm bath, and a stream of cold water to the head." On examination, one of the lower-jaw teeth required scarification, which was done. Nevertheless, the convulsions recurred at short intervals for several hours. It was now ascertained that the child had been feverish for a week previously—the bowels were confined—the abdomen distended—the tongue furred—pulse rapid and skin hot. The head was shaved—six leeches applied to the temples—cold applications to the head. Calomel and antimony, followed by castor-oil. Next day, the convulsions were less frequent; the febrile symptoms mitigated—skin cooler—bowels opened—discharges dark and offensive. The leeches were repeated, and small doses of calomel were continued for the next two or three days. The convulsions ceased, but the fever continued. Stupor came on, with startings and twitchings of the limbs—strabismus and screaming—pulse varying from 160 to 180 in the minute. The child now fell into a torpid state, with all the symptoms of hydrocephalic effusion. In this state, friction with croton oil, and aqua ammoniæ, was employed, and produced a copious pustular eruption on the head and neck. Soon after this, there was a decided amelioration in the symptoms. The torpor became less profound, and sensibility returned. In the course of eight days, the child was out of danger.

This case shews that we ought not to despair of children, when they present the usual symptoms of hydrocephalic effusion. These symptoms, in fact, will all be produced by a turgid state of the cerebral vessels, or an inflamed condition of the membranes—therefore they are not, in all cases, beyond the chance of recovery.

### IV. On the Formation of Hydatids. By Mr. Howship.

The basis of this paper is a case and morbid parts sent to Mr. H. by Mr. Spilsbury, of Walsall, which we shall here insert, together with some remarks by Mr. Howship himself.

"A healthy youth, aged 14 years, had worked as a shoemaker till November, 1830. He said that in 1828, having drunk at a brook, he supposed he had swallowed some spawn of animal growth. On close enquiry, it appeared that he had feverish symptoms, and was frequently sick, presumably from an inflammatory cause; and these symptoms, with the incident thirst, had induced him to drink freely at the brook. The following summer he frequently coughed up blood, and very frequently bled from the nose. His mother spoke of him as a hearty boy previous to drinking at the brook, and said she was herself convinced that he had living animals of the newt kind within him.

During life, from the distinct lobulated feel of the different tumours with which the enlarged abdomen was filled, it appeared probable it might prove to be fungoid disease of some of the viscera, especially as the superficial veins of the abdomen were enlarged and tortuous. Neither the appetite nor the secretions ever failed, with the exception of the bile, with which the skin and urine were highly tinged, the stools being white. The enormously enlarged abdomen measured six feet in circumference; the legs and thighs being loaded with œdema, while the thorax and arms were amazingly extenuated. He died on the 7th November, 1831.

On laying open the abdomen, Mr. Spilsbury found the whole of the viscera one confused mass of tumours, of which a large portion was, by dissection, detached from the right side. In opening the duodenum, a pulpy sac, like coagulable lymph, was cut into, containing probably a full pint of small hydatids, the largest about the size of a hazel-nut. These hydatids were of various tints, from a deep green to a bright yellow, swimming in a thin serous fluid.

The whole of the bowels and other viscera were equally involved in this disease; the continued pressure from which appeared to have very much reduced the size of every viscus by exciting interstitial absorption. The liver, in particular, was diminished in thickness almost to the peritoneal coat, not more than about half an inch of its parenchymatous substance remaining.

The thorax was not inspected, for the limited time afforded did not permit the whole of this extensive disease being unravelled. In the cavity of the abdomen the adhesions were so numerous and intimate that there was neither beginning nor end; the examination, consequently, was only so far satisfactory as the time would permit.

On carefully dissecting the parts forwarded to me, I found that the mass removed from the right side, although composed of different textures, was certainly an entirely new formation. It appeared that acute inflammation had, in the first instance, led to a free effusion of fibrine into the abdomen; that, as the fibrinous matter became organized, new vessels after a time began to secrete serum (at certain points) into the substance of the fibrine; thus inducing the development of serous cysts. These tumours had the appearance of extremely fine thin organized adhesions; and a considerable number of these cysts, some nearly of the size and form of hens' eggs, most of them of a spheroidal figure, and all connected together by extensive fibrinous adhesions, constituted the large mass removed from the right side of the abdomen.

The partial detachment of the external coat, from severel of the tumours, ascertained that each organized, coloured and vascular cyst, contained within it a fine colourless albuminous cyst; devoid of red vessels, and having every character of what Dr. Baillie has termed the true hydatid." 15.

Mr. H. makes a great many other and ingenious observations on the formation and growth of hydatids, for which we must refer to our contemporary of January last.

### V. Rupture of the Tendon of the Biceps Flexor Cubiti. By Sir Geo. Ballingall.

Mr. D. an eminent chemist, aged 50 years, while lifting a heavy weight with the tips of the fingers of the right hand, felt a snap, accompanied by pain in the lower part of the arm, a little above the elbow. The weight dropped from his hand, and there was loss of power in that member. Swelling took place, and there was observed a large tumor at the middle of the arm, occupying the belly of the biceps muscle. A bandage was applied, but when removed the next morning, the nature of the accident was evident. The tendon of the biceps could be felt loose at one extremity, in the hollow left by the retraction of the muscle. Bandages, consisting of two pieces of leather accurately laced—one on the arm and the other on the forearm, with a strap passing from the one to the other, in order to keep the arm bent. The tendon gradually contracted adhesions to the neighbouring parts, and the patient can use his arm tolerably well.

### VI. On the late Influenza in Edinburgh. By Mr. W. Brown.

During the prevalence of the epidemic, the practitioners had something better to do than write about its nature. We need not dwell on it here, as there is not a chemist's apprentice in London who did not become intimately

—indeed personally, acquainted with the epidemic, as it prevailed in 1833. Though simulating, it was something more than a mere catarrh. The catarrhal symptoms were violent, the fever high, and the progress rapid. The debility which followed the attack was most surprising—many people having never yet recovered their former strength. In the bustle and confusion, it was difficult to determine what was the best mode of treatment. Thousands of people had the disease, and violently too, without any remedies at all. They probably fared as well as those who had—"the best advice"—and plenty of physic into the bargain! Those who kept to their beds, and took abundance of diluents, so as to promote perspiration, got through the epidemic best. There was something so debilitating about the disease, that bleeding was seldom necessary except in particular constitutions, where the inflammatory symptoms ran very high—and more especially where bronchitis continued after the epidemic itself had vanished. The sequelæ of the influenza, indeed, were infinitely worse than the epidemic itself—and many thousands have since died of these sequelæ. All the evidences of contagion in cholera were here ten times stronger, and yet very few medical men will stand up for contagion in influenza. Cullen, and, indeed, our Caledonian brethren generally, have evinced an itch for contagion beyond those of most other countries, as may be seen by the great nosologist placing a series of epidemics under the title of "*catarrhus a contagio*," and the great expense of gunpowder and vinegar which our neighbours incurred during the epidemic cholera, by way of destroying the virulent infection of that highly communicable disease. Our present author, however, is a very moderate contagionist in influenza—indeed he may be ranked amongst the contingent contagionists. The immediate mortality of the disease in Edinburgh was not considerable. It was in its sequelæ that it was formidable.

### VII. Epidemic Scarlatina—Heriot's Hospital. By Mr. Wood.

This epidemic occurred in the last three months of 1832. The establishment contained 179 boys, of whom 44 became affected with the disease. Thirty-three out of the 179 were supposed to have had the disease previously —and 5 out of the 44 were considered second attacks. The fever could not be traced into the hospital, and it occurred while the establishment was under strict quarantine to keep out cholera! The epidemic was a mild one, and only one patient died out of the 44. The fever ran pretty high, and delirium was not unfrequent. In all cases there was more or less affection of the throat, though in some it was very slight. In none of the cases was there any extensive sloughing. The treatment was simple. Purgative medicines were exhibited daily till the fever subsided, and continued at intervals afterwards till the patient was considered cured. The purgatives were compound powder of jalap, epsom salts—colocynth, senna, &c. The patients were kept moderately cool, and the rooms well ventilated. During the eruptive fever, when the skin was very hot, the surface was sponged, sometimes with cold, sometimes with warm water. Great relief was derived from this practice. The patients were kept on the lowest scale of diet, unless the fever assumed a typhoid form, when some wine was allowed. Nine out of the 44 cases were followed by dropsical effusion. The average appearance of the effusion was nineteen days after the commencement of the fever. The one fatal case was from the dropsy. On dissection, a great deal of vinous engorgement appeared in the brain, and considerable serous effusion had taken place between the membranes and in the ventricles. There was but a small quantity in the abdomen. This dissection is not given from the author, but from a long paper reprinted from Dr. Hamilton's Work, relative to an epidemic of the same kind in 1804.

### VIII. RECOVERY FROM SUSPENDED ANIMATION. By Dr. M'WHIRTER.

Dr. M'W. offers this case by way of encouragement to his obstetric brethren, " who must occasionally meet with cases of new-born infants *suffering* from suspended animation." We confess that this is a new feature in physiology—*suffering* from suspension of life. Be this as it may, the Doctor used every energetic measure for restoring sensibility to his little patient, and, by bringing it into a troublesome world, has, we fear, been the innocent cause of much more *suffering* than it experienced during the *humane* process of the resuscitator. After the common means were used for nearly half an hour, without success, and after " nearly two bottles of brandy and whiskey had been expended on frictions," &c. besides " slapping the bottom" occasionally, a sob was observed—but it was nearly an hour and a half before " the child gave a whimper." The Doctor deserves a laurel crown—and we think Malthus himself, could he look from the grave, would say AMEN.

A very able paper of 20 pages, on " Thoracic Pathology," by assistant-surgeon Poole (32d regiment), follows the Medico-Chirurgical Transactions, and we wish that our limits would permit us to notice it at some length. It consists, however, of cases minutely detailed, and which we could not condense or analyze. The case of gangrene of the lungs, following an attack of cholera, and where there was collapse, with absence of pulse for 24 hours, is exceedingly interesting, and, indeed, the whole paper is very creditable to Mr. Poole.

